# Chronic Inflammation and Altered Immune Responses in LongCOVID Associate with Neurological Manifestations and Accelerated Aging

**DOI:** 10.3390/cells14231875

**Published:** 2025-11-26

**Authors:** Norina Tang, Judith M. Ford, Kaitlyn Dal Bon, Lynn Pulliam

**Affiliations:** 1 Department of Laboratory Medicine, San Francisco VA Health Care System, San Francisco, CA 94121, USA; norina.tang@va.gov; 2 Department of Mental Health, San Francisco VA Health Care System, San Francisco, CA 94121, USA; judith.ford@ucsf.edu (J.M.F.); kaitlyn.dalbon@ucsf.edu (K.D.B.); 3 Department of Laboratory Medicine, University of California, San Francisco, CA 94143, USA

**Keywords:** longCOVID-19, PASC, plasma biomarkers, brain fog, Ella automated microfluidic ELISA, inflammaging, accelerated aging

## Abstract

There is a subgroup of people infected with the SARS-CoV-2 virus who manifest lingering sequelae (LongC), with neurological symptoms (nLongC). We recruited 86 COVID-19 volunteers, 35 of whom were fully recovered (Cov) and 51 who had neurological symptoms (nLongC) 4–53 months after infection and compared them to 51 healthy pre-pandemic controls (HC). Thirty-five percent of nLongC individuals carried the apolipoprotein E4 (APOE4) gene, compared to 11% of Cov. Four plasma proteins, interleukin 1 beta (IL-1β), interleukin 8 (IL-8), glial fibrillary acidic protein (GFAP), and hemopexin, continued to be elevated in both Cov and nLongC compared to HC. Soluble CD14 was elevated in nLongC but not Cov. As a group, IL-1β decreased over time in Cov but not nLongC. Two of the elevated proteins, IL-8 and GFAP, correlated with age, with both Cov and nLongC showing higher levels than HC. Using a combination of four plasma proteins, along with age, body mass index, and APOE4 presence, we were able to achieve an area under the curve (AUC) of 0.81. These results suggest that SARS-CoV-2 infection causes a low-grade inflammatory process that, even months or years after infection, does not return to pre-COVID-19 levels, which may contribute to neurologic sequelae and accelerated aging.

## 1. Introduction

It has been over 5 years since the COVID-19 pandemic began. As the SARS-CoV-2 virus continues to evolve and mortality declines steadily, vaccination rates have gone down, and reporting of mild infections has curtailed. A World Health Organization-led clinical case definition of longCOVID-19 (LongC), also called post-acute sequelae of COVID-19 (PASC), long-haul COVID-19 or post-COVID-19 condition (PCC), has evolved to be a condition that lasts at least 2 months after SARS-CoV-2 infection, involving a myriad of symptoms that cannot be explained by another diagnosis [1]. Global prevalence of LongC can occur in with mild-to-moderate infection as well as with serious illness [2,3,4]. Since reporting of LongC is no longer consistent, statistics are based on early studies showing the prevalence of LongC in nonhospitalized people as 34% and worldwide as 54% among those who required hospitalization [5].

Because there are numerous symptoms of LongC, an agreed upon mechanism for the condition is elusive. The number of individuals affected is unknown, since this was a worldwide pandemic and access to care was not uniform. Cognitive dysfunction or “brain fog”, also called neuroLongC (nLongC), continues to be the most frequent symptom after fatigue and can appear later in the illness [5,6,7]. Diagnostic markers for LongC or nLongC have not been reported. The effect of a long-term symptomatic course of LongC not only influences lifestyle but may have major impact on accelerating age-related neurodegeneration. Early studies in the pandemic were focused on lingering symptoms in relatively older and hospitalized individuals. The concern was that chronic inflammation in older individuals has significant risks for neurodegeneration [8], since neuroinflammation alone can cause cognitive dysfunction [9,10]. Presently, there are younger-to-middle-aged individuals with complaints of “brain fog” months to years after an uneventful infection with SARS-CoV-2, leading to concerns about lasting neurological effects from LongC in these otherwise healthy individuals in the prime of their lives [11].

The goals of this exploratory, cross-sectional study were to determine the contribution of apolipoprotein E4 (APOE4) gene presence, identify plasma proteins associated with cognitive impairment, and validate our findings with another platform that has clinical utility. We evaluated a panel of cytokines that we previously published on nLongC, which were upregulated months after infection [12], and added other soluble markers from the literature on COVID-19 to determine a predictive panel of markers that persist over time and may explain the lingering sequelae. We recruited people with nLongC with symptoms lasting longer than 4 months post infection, which included new or worsening neurological symptoms or “brain fog” by self-report. In the nLongC group, we found that interleukin 1 beta (IL-1β), interleukin 8 (IL-8), soluble cluster of differentiation 14 (CD14), glial fibrillary acidic protein (GFAP), and hemopexin (HPX) were all elevated in the plasma. Even in the COVID-19-recovered (Cov) group, who had no lingering symptoms, we found markers associated with aging that were elevated early and persisted months to years after infection despite symptom resolution. Soluble CD14, a monocyte activation marker associated with cognitive impairment, brain aging, and atrophy [13], was the only analyte tested to be significantly elevated in nLongC compared to Cov and healthy controls (HCs). IL-1β, a cytokine that mediates neuroinflammation, was elevated in both Cov and nLongC, but decreased over time in Cov and not in nLongC. Using these plasma markers, associated with altered immune responses, inflammaging, and cognitive impairment, along with APOE4, age, and body mass index (BMI), we were able to identify nLongC with over 80% accuracy.

## 2. Materials and Methods

### 2.1. Study Participants and Blood Collection

Eighty-six volunteers with a documented history of SARS-CoV-2 virus infection, as evidenced by a positive viral RNA PCR or antigen test result from a nasal or throat swab, were recruited from the San Francisco Veterans Affairs Health Care System and from advertising through social media in the San Francisco Bay Area. All were 4–53 months post SARS-CoV-2 infection, and all signed a written informed consent approved by the University of California, San Francisco Institutional Review Board. Of the 86 total volunteers, 35 had recovered from SARS-CoV-2 infection with no residual symptoms (Cov), while 51 volunteers had neurological complaints (nLongC) consisting of at least one of the following: difficulty with memory or concentration, and increased anxiety or depression. Exclusion criteria for study participation included past and present seizures, head trauma, loss of consciousness > 15 min, alcohol and/or substance abuse/dependence within 3 months of participation, history of human immunodeficiency virus (HIV) infection, and current pregnancy. Fasting whole blood was collected in ethylenediaminetetraacetic acid (EDTA) tubes between February 2022 and November 2024. Plasma and peripheral blood mononuclear cells (PBMCs) were collected and frozen in aliquots at −80 °C as indicated below.

For the HC cohort, we used 51 frozen plasma samples purchased from Blood Centers of the Pacific (Vitalant, San Francisco, CA, USA) that were collected from pre-pandemic individuals. Purchased frozen plasma for the HC cohort was also aliquoted and stored at −80 °C until use.

### 2.2. Plasma Collection

Fasting EDTA blood was centrifuged at 300× *g* without brake at room temperature (RT) for 15 min, and the supernatant above the interface was transferred to fresh tubes. The collected plasma was then centrifuged at 300× *g* with brake at 4 °C for 5 min, and the supernatant was collected and subjected to a final centrifugation at 1000× *g* at 4 °C for 10 min. Spun plasma from the same individual were pooled, mixed, aliquoted, frozen, and stored at −80 °C until use.

### 2.3. Peripheral Blood Mononuclear Cell (PBMC) Collection

Fasting EDTA blood was diluted 1:1 with PBS containing 2 mM EDTA (PBS/EDTA). Approximately 25 mL of diluted blood was layered onto 12.5 mL LymphoPrep™ density gradient medium (STEMCELL Technologies Inc., Cambridge, MA, USA, catalog # 18061) and centrifuged at 400× *g* without brake at RT for 20 min. After centrifugation, PBMCs located at the interface of the plasma and LymphoPrep™ layers were collected using a pipette. Subsequently, the PBMCs were first washed by bringing the volume up to 50 mL with PBS/EDTA followed by centrifugation at 300× *g* without brake at 4 °C for 5 min. After gently removing and discarding the supernatant, the PMBCs were washed a second time by resuspending the spun PBMC pellet in 50 mL PBS/EDTA followed by centrifugation at 200× *g* without brake at 4 °C for 10 min. Afterwards, the PBMC pellet was resuspended in 10 mL cold PBS/EDTA, and the cell suspension was counted using a hemacytometer.

For the APOE genotyping assay below, 2 × 10^6^ PBMCs were placed into a microfuge tube and centrifuged at 300× *g* without brake at 4 °C for 5 min. After discarding the supernatant, the cell pellet was resuspended and lysed with 350 mL of RLT Lysis Buffer (Qiagen, Germantown, MD, USA, catalog # 1053393). Lysed PBMCs were stored frozen at −80 °C until use.

Remaining washed PBMCs were centrifuged at 300× *g* without brake at 4 °C for 5 min, and the supernatant was discarded. The PBMC pellet was gently resuspended to a final concentration of 1 × 10^7^ cells/mL using cold freezing media (10% DMSO in fetal bovine serum), aliquoted, and stored in liquid nitrogen.

### 2.4. APOE Genotyping

DNA was isolated from the frozen, lysed PBMCs (prepared as indicated above) using the AllPrep DNA/RNA Mini kit (Qiagen, Germantown, MD, USA, catalog # 80204). The isolated PBMC DNA was added to TaqMan™ Fast Advanced Master Mix reagents (Thermo-Fisher Scientific, Inc., Waltham, MA, USA, catalog # 4444554), amplified, and subjected to TaqMan™ SNP Genotyping Assays for rs429358 and rs7412 (Thermo-Fisher Scientific, PN 4351379) using the ABI ViiA 7 (Applied Biosystems Co., Waltham, MA, USA) instrument for endpoint PCR. Data were analyzed using the TaqMan™ SDS software version 1.2.1 (Thermo-Fisher Scientific, Inc.). For quality control, positive and negative controls were included in all PCR assays. The negative control consisted of water (i.e., no DNA) while the positive control consisted of DNA for the ε3/ε4 genotype (Coriell Institute for Medical Research, Camden, NJ, USA, catalog # NG11755).

Because the APOE gene is polymorphic at the two nucleotides rs429358 and rs7412, six genotypes are possible: ε2/ε2, ε2/ε3, ε2/ε4, ε3/ε3, ε3/ε4, and ε4/ε4 [14].

### 2.5. Plasma Cytokine Analysis Using Meso Scale Discovery (MSD) Assays

For a subset of the HC, Cov, and nLongC cohorts, a multiplex assay of seven plasma cytokines (IL-1β, IL-4, IL-6, IL-8 (CXCL8), IL-10, TNFα, and IFNγ) were measured using the V-PLEX Viral Panel 2 Human Kit multiplex assay from MSD (Rockville, MD, USA, catalog # K15346D-1). Dynamic ranges for these seven analytes are as follows: IL-1β, 0.05–375 pg/mL; IL-4, 0.02–158 pg/mL; IL-6, 0.06–488 pg/mL; IL-8, 0.07–375 pg/mL; IL-10, 0.04–233 pg/mL; TNFα, 0.04–248 pg/mL; and IFNγ, 0.37–938 pg/mL.

Additional plasma samples were analyzed for IL-1β, IL-8, and IL-10 using a custom VPLEX duplex assay from MSD for IL-1β and IL-8 (catalog # K151ARH-1), and a V-PLEX single-plex assay for IL-10 (catalog # K0082525). These assays use the same antibodies and reagents as the 7-plex assay above and, thus, have the same sensitivities, allowing us to pool the data.

IL-18 levels were queried using a U-PLEX single-plex assay from MSD (catalog # K151VJK-1). The detection range for IL-18 is 0.5–14,000 pg/mL.

Soluble CD14 and soluble CD163 levels were quantified using a custom R-PLEX duplex assay from MSD (catalog # K15227N-1 with CD14 antibody set catalog # F21T1-3 and CD163 antibody set catalog # F21J4-3). The lower limit of detection is 1.3 pg/mL for CD14 and 77 pg/mL for CD163.

Complement C3 (C3) and HPX levels were quantified using a custom R-PLEX duplex assay from MSD (catalog # K15227N-1 with C3 antibody set catalog # F21XY-3 and HPX antibody set catalog # F217F-3). The lower limit of detection is 770 pg/mL for C3 and 0.90 pg/mL for HPX.

All MSD assays were performed in duplicate following manufacturer’s instructions. Results were read using the QuickPlex SQ 120 instrument (MSD), and analyses were performed using DISCOVERY WORKBENCH^®^ 4.0 software (MSD).

### 2.6. Fibrinogen Quantification

Fibrinogen levels in the plasma were quantified at the San Francisco VA Health Care System, Laboratory Medicine Service, Hematology Clinical Laboratory, using the STA R Max coagulation analyzer (Diagnostica Stago, Inc., Parsippany, NJ, USA).

### 2.7. Plasma Neural Protein Analysis Using High Sensitivity MSD Assays

MSD S-PLEX single-plex assays were used to quantify the GFAP (catalog # K151AMPS) and neurofilament light chain (NFL, catalog # K151AKGS) levels in plasma. The detection ranges are 0.150–1100 pg/mL for GFAP and 1.7–1400 pg/mL for NFL. Assays were performed in duplicate following the manufacturer’s instructions, and results were read using the QuickPlex SQ 120 instrument (MSD). DISCOVERY WORKBENCH^®^ 4.0 software (MSD) was used for the results analysis.

### 2.8. Validation Using Ella Automated ELISA System

Ella^TM^, the automated ELISA system available from Bio-Techne (Newark, CA, USA) was used to measure plasma IL-1β, IL-8, CD14, and GFAP using customized, multiplex 32 × 4 cartridges. Plasma samples were diluted 2-fold using SD13 sample diluent supplied by the manufacturer. A total of 50 μL of diluted plasma, along with manufacturer-supplied Wash Buffer A (Bio-Techne), were loaded into their respective wells of the cartridge. The cartridge was then placed into the Ella instrument for testing. The run time on the Ella instrument was approximately 90 min. The Ella instrument performs the assay in triplicate. Accepted data must have relative fluorescent unit values with a <10% coefficient of variance (CV). Reported results represent the mean of accepted values (triplicates or duplicates with a <10% CV). Quantitative ranges for the four analytes are as follows: IL-1β, 0.4–1530 pg/mL; IL-8, 0.19–1804 pg/mL; CD14, 16.92–4130 pg/mL; and GFAP, 2.52–9600 pg/mL.

### 2.9. Statistical Analysis and Bioinformatics

Statistical analyses were performed using GraphPad Prism 10 software (GraphPad Software Inc., Boston, MA, USA). Our data were not normally distributed. Therefore, when comparing differences between 3 or more groups, the Kruskal–Wallis test was used followed by post hoc Dunn’s test to correct for multiple comparisons. When comparing the non-normally distributed data between 2 groups, the two-tailed Mann–Whitney test was used. Group differences in categorical data were assessed using Fisher’s exact test. * denotes *p* ≤ 0.05, ** denotes *p* ≤ 0.01, *** denotes *p* ≤ 0.001, and **** denotes *p* ≤ 0.0001. *p* values ≤ 0.05 were considered significant. Associations between quantitative variables were determined using a two-tailed Spearman correlation test along with simple linear regression to illustrate trend lines. Multivariate logistic regression analyses using the main effects model were performed to determine the odds ratio of each of the risk factors and predict nLongC.

Gene enrichment and functional annotation analyses were performed using Database for Annotation, Visualization, and Integrated Discovery (DAVID) [15] and g:Profiler, version e112_eg59_p19_25aa4782 with the g:SCS multiple-testing correction method applying a significance threshold of 0.05 [16,17].

## 3. Results

### 3.1. Particicpant Demographics, Clinical Measures and APOE Genotype

**Figure 1:**
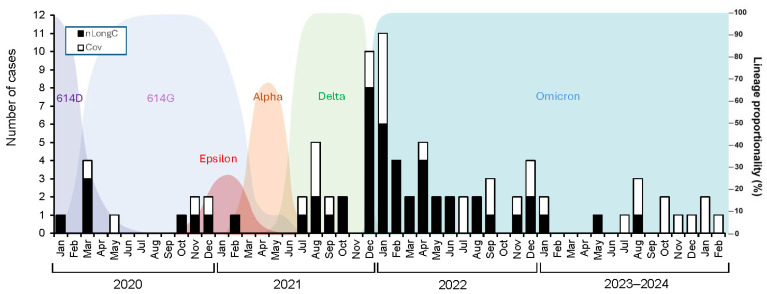
Infection dates of the COVID-19 study participants. The 86 COVID-19 cases described in this study (nLongC and Cov) were infected over a period of 4+ years with SARS-CoV-2. Although not formally genotyped, these 86 participants were, most likely, infected with different viral variants. The number of nLongC cases for each month is shown with black bars, while Cov cases are shown with white bars, all superimposed onto a background of SARS-CoV-2 variants prevalent at the time [22,23,24]. Four nLongC and 2 Cov participants were infected more than once, but only the initial/first infection date is plotted here.

We recruited 86 people who tested positive by a nasal or throat swab for SARS-CoV-2 using a nucleic acid PCR test or rapid antigen test. These individuals were most likely infected with different viral variants (Figure 1) and were assessed 4–53 months post infection. None were hospitalized. Of the 86, 35 did not report any lingering symptoms from infection, while 51 expressed concerns of lingering or worsening symptoms including neurologic complaints (Table 1). For comparisons, plasma from 51 HC individuals were obtained from the local blood bank between 14 July 2019–20 February 2020.

**Figure 2:**
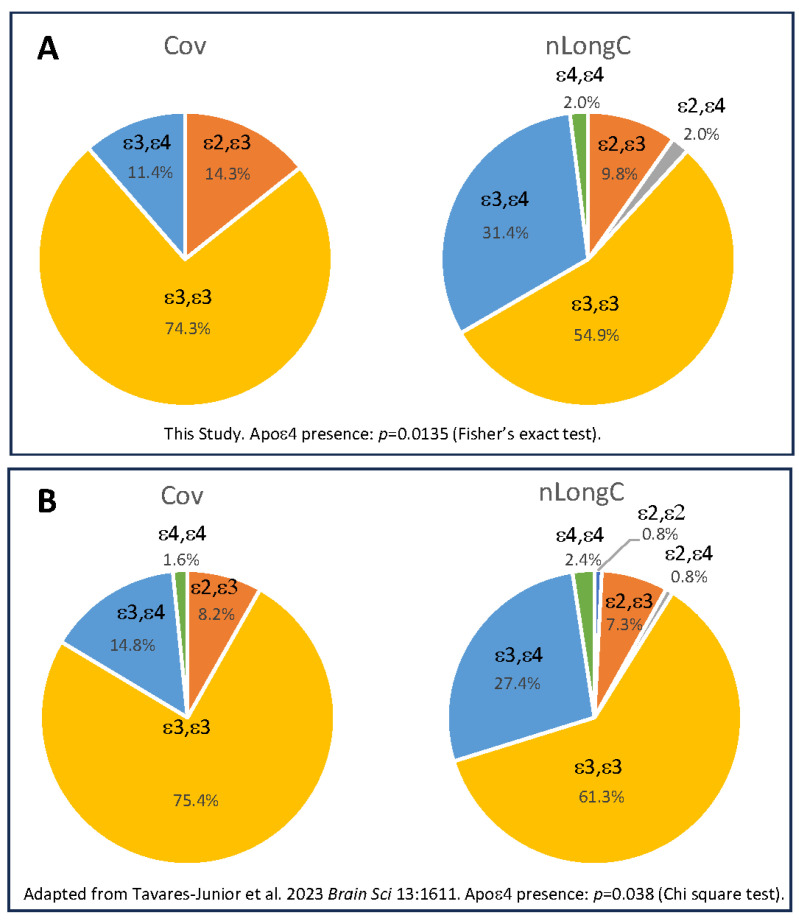
APOE frequencies between cohorts. (**A**) People with nLongC were significantly more likely to have the APOE4 gene. These results aligned with a larger cohort published previously by a different group, (**B**) (adapted from [18]).

Most of the participants were white. There was a significant difference in the presence of the APOE4 gene between the Cov and nLongC participants, with 35% of the nLongC participants having the ε4 allele compared to 11% of the Cov group (Figure 2A). While our numbers are small, a much larger study of 219 people reported that 65.3% had cognitive decline post infection, and of those, 30.6% had the APOE e4 allele versus 16.4% for the cognitively normal group (Figure 2B) [18]. Our nLongC group had significantly more co-morbidities than the recovered Cov cohort. We included BMI, as several reports showed that obesity was associated with more severe COVID-19 infection, as well as LongC [19,20,21]. In this study, there was not a significant difference in BMI between Cov and nLongC. Older age has also been reported to be a risk factor for nLongC [19]. In our three cohorts, the age ranges were similar and more likely considered middle-aged. There were also no significant differences in mean age between the groups (HC-nLongC, *p* = 0.22; Cov-nLongC, *p* = 0.46) except that the Cov group was significantly younger than the HC group (*p* = 0.007).

### 3.2. Cytokine and Plasma Biomarkers for Peripheral Inflammation, Neuroinflammation and Vascular Health

**Figure 3:**
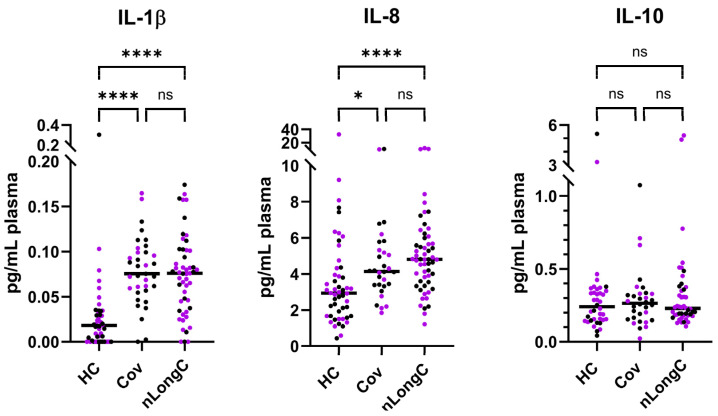
Plasma cytokine levels. There was a significant increase in plasma cytokines IL-1β and IL-8 in Cov and nLongC compared to HC, but not between Cov and nLongC. IL-10 showed no difference between the groups. Purple dots represent previously published results [12], and black dots represent additional new sample data. For IL-1β and IL-8, the total sample sizes were 51 HCs, 35 Cov, and 51 nLongC. Due to limited plasma availability, the sample sizes for the IL-10 analyses were reduced to 38 HCs, 32 Cov, 46 nLongC. Horizontal bars indicate group medians, which were compared using Kruskal–Wallis tests, followed by post hoc Dunn tests to correct for multiple comparisons. ns = *p* > 0.05, * *p* ≤ 0.05, and **** *p* ≤ 0.0001.

IL-1β is a potent pro-inflammatory cytokine and when chronically expressed can contribute to several inflammatory diseases. We previously published, using a smaller cohort 5–24 months post infection, that there was a significant increase in IL-1β between HC and Cov, and between HC and nLongC [12]. These previously published results are shown in Figure 3 as purple dots (28 HC, 12 Cov, 33 nLongC), with additional participants measured in this study at 4–53 months post infection shown as black dots (23 HC, 13, Cov, 18 nLongC). Pooling the data, we see that IL-1β continues to be significantly elevated in both Cov and nLongC.

IL-8, also called CXCL8, is a pro-inflammatory cytokine predominantly secreted by macrophages, which can also act as a chemokine in infection. With the added samples, IL-8 continued to be elevated in nLongC compared to HCs, with a more significant difference than previously observed [12]. IL-10 is an anti-inflammatory cytokine that was trending up in nLongC in our previous report [12]. With added participants, there were still no differences between the groups. Due to limited plasma availability, the sample sizes for the IL-10 analyses were reduced (38 HCs, 32 Cov, and 46 nLongC).

**Figure 4:**
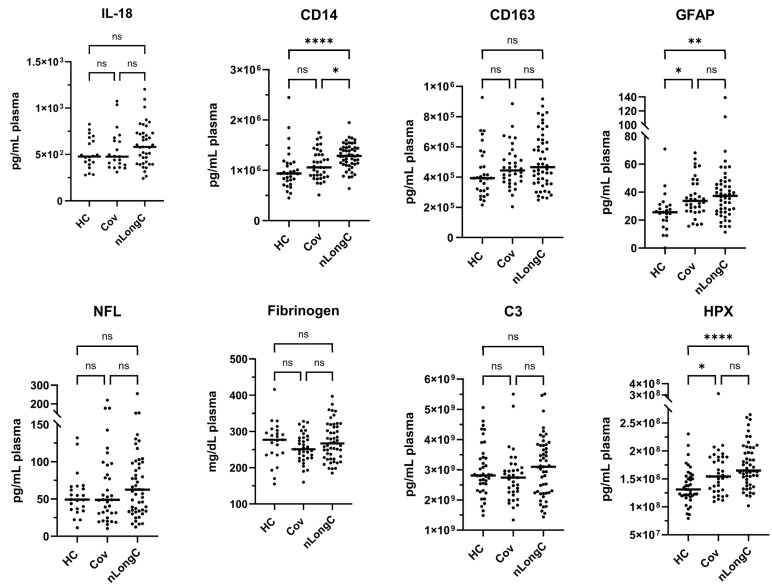
Plasma markers of peripheral inflammation, neuroinflammation, and vascular health. There were significantly elevated levels of CD14, GFAP, and HPX in nLongC compared to HCs. Only CD14 levels were higher in nLongC compared to Cov individuals. All assays were performed using the MSD platform. Due to sample availability, cohort sizes varied between the assays as follows: IL-18 (20 HCs, 21 Cov, 39 nLongC); CD14 and CD163 (30 HCs, 35 Cov, 51 nLongC); GFAP, NFL, and fibrinogen (23 HCs, 35 Cov, 51 nLongC); and C3 and HPX (40 HCs, 35 Cov, 51 nLongC). Horizontal bars indicate group medians, which were compared using Kruskal–Wallis tests, followed by post hoc Dunn tests to correct for multiple comparisons. ns = *p* > 0.05, * *p* ≤ 0.05, ** *p* ≤ 0.01, and **** *p* ≤ 0.0001.

Based on the LongC literature, we analyzed additional plasma targets to measure inflammation, neuroinflammation, and vascular health. These assays were performed using single-plex or duplex MSD assays, and the number of samples varied between analytes because of sample volume limitations. IL-18 is a pro-inflammatory cytokine in the IL-1 family and is associated with host defense. It is elevated in acute COVID-19 infection and a marker of disease severity [25]. An increase in IL-18 after COVID-19 infection correlated with inflammation and severe disease [26]. We did not see a significant difference in the levels of IL-18 between the three groups (20 HCs, 21 Cov, and 39 nLongC) (Figure 4).

Soluble CD14 and soluble CD163 are both associated with macrophage activation. CD14 was previously reported to be significantly elevated in LongC compared to HC and recovered COVID-19 individuals [27]. Like the previous report [27], we found in our cohort that CD14 was elevated in nLongC compared to both Cov and HC groups. CD14 was the only plasma biomarker we tested that differentiated the Cov from the nLongC cohort (Figure 4). CD163 has been reported to be increased in hospitalized COVID-19 patients compared to non-hospitalized COVID-19 individuals and healthy controls [28,29]. In this study, CD163 showed no statistical differences between the groups (Figure 4).

GFAP and NFL are both markers of neuroinflammation and potential biomarkers for early diagnosis of cognitive impairment and dementia [30]. Both soluble GFAP and NFL have previously been reported to be elevated in LongC and nLongC [31]. Both normally increase with age [32,33,34,35], and correlate with risk of Alzheimer’s disease (AD) [33]. In this study, plasma GFAP was significantly elevated in both Cov and nLongC compared to HCs (Figure 4). Although a previous report showed that people with LongC and neurocognitive symptoms had significantly higher levels of NFL compared to healthy controls [36], we did not observe such differences in our cohort (Figure 4).

Thromboinflammation is a possible mechanism for the inflammation and neurologic consequences of LongC [37,38]. Certainly, in the acute phase of COVID-19, coagulation abnormalities contributed to the severity of the infection [39]. Increased fibrinogen was reported in hospitalized COVID-19 patients, correlating with severity of disease [40,41]. In this study, we found no differences in the levels of fibrinogen between the HC, Cov, and nLongC groups (Figure 4). Complement C3 has also been reported as a marker of COVID-19 severity and progression [42,43,44], but we did not find differences in C3 between the three groups (Figure 4). HPX is a molecule that is released during red blood cell breakdown and binds to free heme, preventing harm and the initiation of inflammation. Plasma HPX increases with inflammation and was reported to be higher in hospitalized COVID-19 patients compared to controls [45,46]. HPX is also implicated in AD with increases in the CSF of AD patients compared to cognitively normal subjects [47]. Elevated plasma HPX levels were associated with increased brain amyloid deposition as measured by PET neuroimaging using ^11^C-PIB [48]. We found a significant increase in plasma HPX in nLongC and Cov compared to HCs (Figure 4).

### 3.3. Correlation Matrices and Patterns

**Figure 5:**
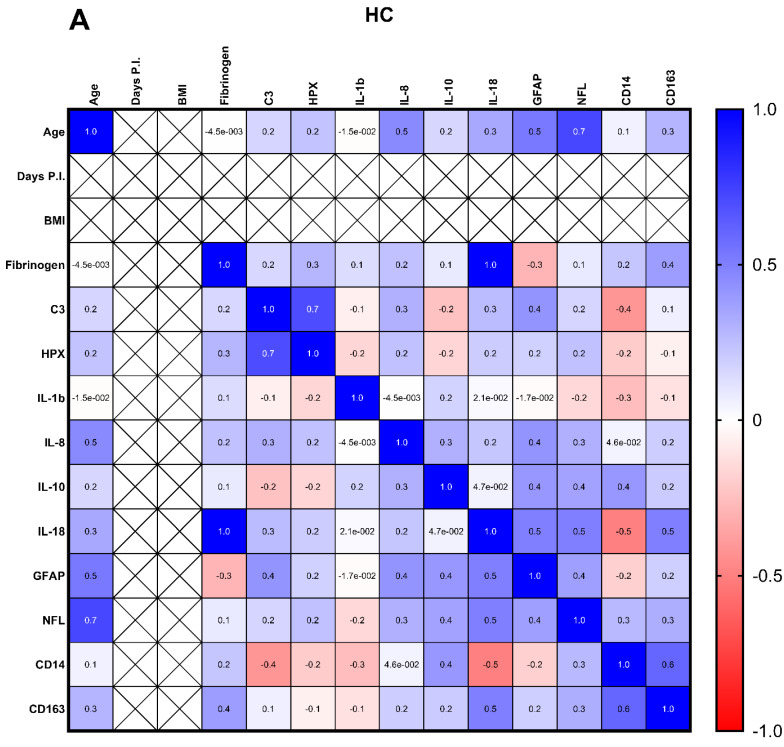

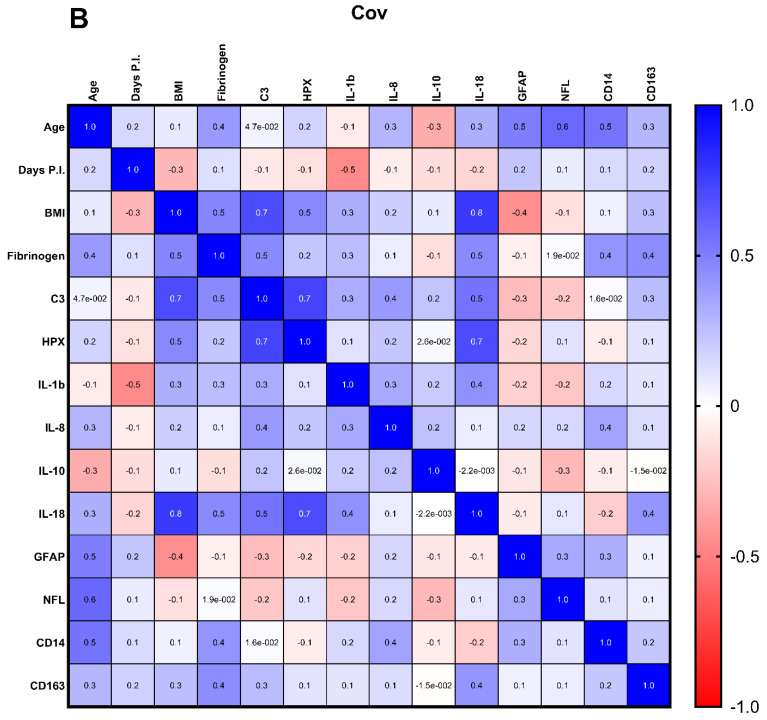

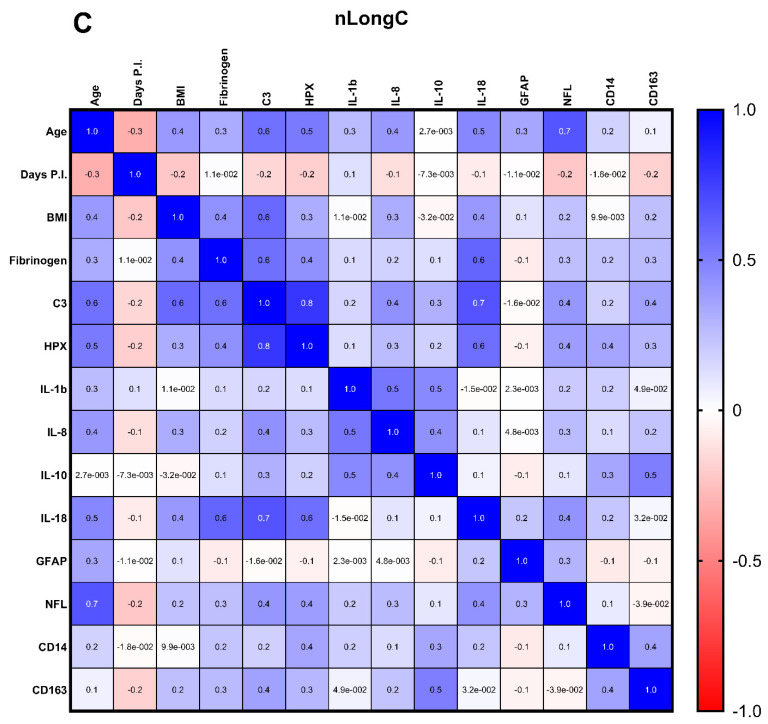
Multi-parameter correlation matrices for (**A**) HC, (**B**) Cov, and (**C**) nLongC. The number in each square is the Spearman rank correlation coefficient. Positive correlations are in blue, and negative correlations are in red. The magnitude of correlation is illustrated by the color intensity, as shown by the scale bar to the right. *p* values for the correlations can be found in Table A1. Group sample sizes for each of the variables are as follows: age, IL-1β, and IL-8: 51 HCs, 35 Cov, and 51 nLongC; days P.I. and BMI: 35 Cov and 51 nLongC; IL-10: 38 HCs, 32 Cov, and 46 nLongC; IL-18: 20 HCs, 21 Cov, and 39 nLongC; CD14 and CD163: 30 HCs, 35 Cov, and 51 nLongC; GFAP, NFL, and fibrinogen: 23 HCs, 35 Cov, and 51 nLongC; and C3 and HPX: 40 HCs, 35 Cov, and 51 nLongC.

The relationships between each of the clinical measures, APOE4 status, and analytes investigated were plotted using a correlation matrix. The overall pattern of relationships was different between the three groups, with nLongC showing more positive correlations between the markers than HCs and Cov (Figure 5).

### 3.4. Chronic Inflammation and Altered Immune Responses in nLongC

**Figure 6:**
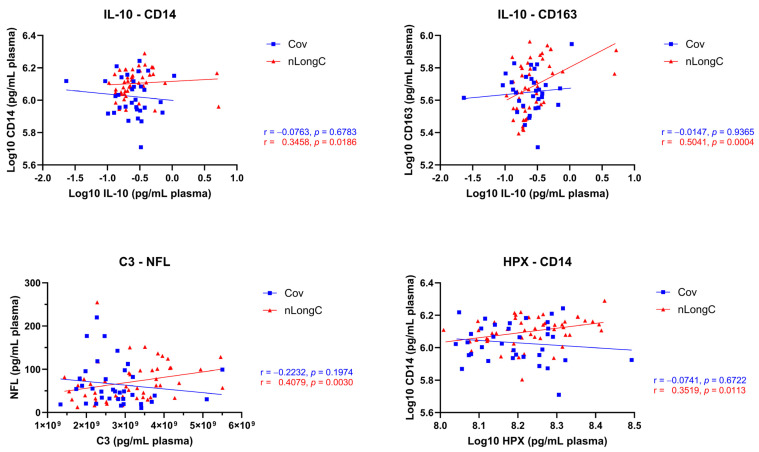
Protein correlation graphs show different trajectories between Cov and nLongC cohorts, suggesting altered immune response in nLongC. Individuals with nLongC had significant positive correlations with elevated slopes compared to recovered individuals who showed no significant correlations and negative slopes. Shown are Spearman rank correlation coefficients (r) along with their corresponding two-tailed *p* values. Group sample sizes for each of the variables are as follows. IL-10: 38 HCs, 32 Cov, and 46 nLongC; CD14 and CD163: 30 HCs, 35 Cov, and 51 nLongC; NFL: 23 HCs, 35 Cov, and 51 nLongC; and C3 and HPX: 40 HCs, 35 Cov, and 51 nLongC.

A closer examination of correlation relationships (Figure 5) revealed immune responses that were different between Cov and nLongC despite insignificant differences in overall levels for some of these markers (Figure 3 and Figure 4). nLongC showed positive correlations between IL-10 and CD14, between IL-10 and CD163, between C3 and NFL, and between HPX and CD14 (Figure 6). In contrast, Cov did not show positive relationships, perhaps even showing negative correlations. These relational differences suggest a dichotomous immune regulation with one, possibly, leading to recovery from COVID-19 and the other to chronic inflammation and neurological sequelae.

**Figure 7:**
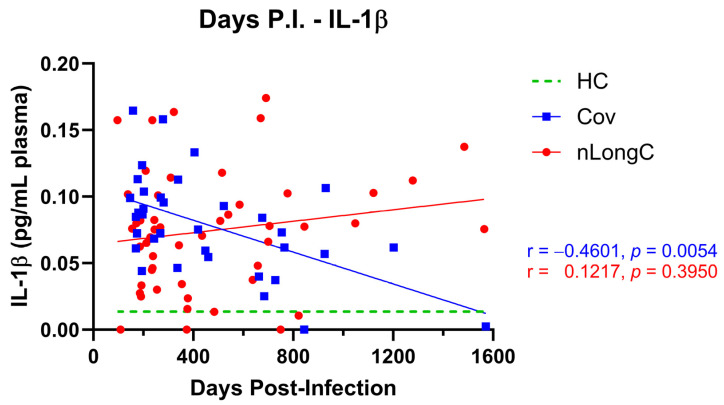
IL-1β levels over time. As a group, IL-1β decreased over time in Cov but not in nLongC. The green dotted line represents the median value of HCs (0.01351 pg/mL plasma). r = Spearman rank correlation coefficient; *p* = two-tailed *p* value. Group sample sizes: 51 HCs, 35 Cov, and 51 nLongC.

**Figure A1:**
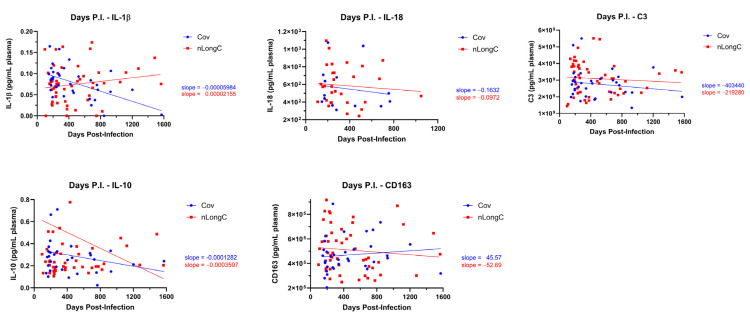
Plasma protein levels over time post infection. As a group, inflammatory proteins IL-1β, IL-18, and complement C3 decreased at a faster rate in Cov versus nLongC. In contrast, anti-inflammatory proteins IL-10 and CD163 decreased at a faster rate in nLongC compared to Cov. Slopes are displayed.

When we looked at changes in analytes as a group over time after infection, the most dramatic change was in IL-1β (Figure 7). As a group (although not individually as this is not a longitudinal study), there was a moderate negative correlation with IL-1β in people who recovered from COVID-19 (r = −0.46, *p* = 0.005), while in people with lingering neurological sequelae, IL-1β did not decrease over time (r = 0.12, *p* = 0.395). There were also differences in the slopes of the simple linear regression lines between other proteins (IL-18, C3, IL-10, and CD163) and time post infection that further highlighted the dissimilarity between Cov and nLongC immune recovery (Figure A1). Inflammatory proteins IL-1β, IL-18, and complement C3 decreased at a faster rate in Cov, while anti-inflammatory proteins IL-10 and CD163 decreased at a faster rate in nLongC.

### 3.5. Association of Protein Markers to Aging

**Figure 8:**
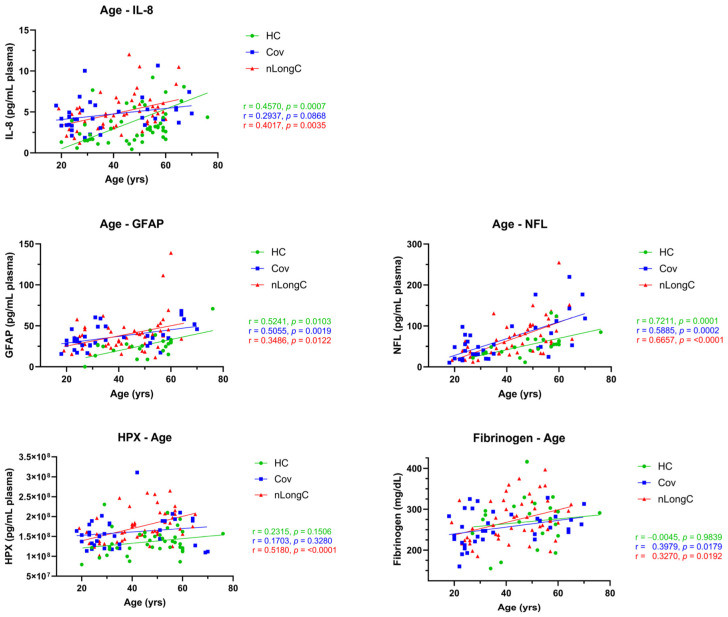
Correlations of age with IL-8, GFAP, NFL, HPX, and fibrinogen. When adjusted for age, IL-8, GFAP, NFL, HPX, and fibrinogen were all increased in nLongC. Three of these, GFAP, NFL, and fibrinogen, were also increased in Cov. Shown are Spearman rank correlation coefficients (r), along with their corresponding two-tailed *p* values. Group sample sizes for each of the variables are as follows: age and IL-8: 51 HCs, 35 Cov, and 51 nLongC; GFAP, NFL, and fibrinogen: 23 HCs, 35 Cov, and 51 nLongC; and HPX: 40 HCs, 35 Cov, and 51 nLongC.

Age is a risk factor for severe COVID-19 infection [49,50]. SARS-CoV-2 infection is associated with premature or accelerated epigenetic aging such as DNA methylation and telomere length [51,52]. Another type of accelerated aging is driven by chronic inflammation and is called inflammaging [53]. Given that the participants in this cohort were middle-aged with a mean age of 38 years for Cov and 43 years for nLongC, we asked whether SARS-CoV-2 infection promoted inflammaging by correlating age-related plasma proteins to age. We found that IL-8 significantly increased with age in HCs (Figure 8), consistent with a recent report by another group [54]. In Cov and nLongC individuals, IL-8 levels were further elevated, even after age adjustment, until around 60 years old (Figure 8).

GFAP and NFL are markers of neuroinflammation that rise with age [34,55,56,57,58,59], increase in a severity-dependent manner during COVID-19 infection [60], and are elevated in people with persistent neurological complications of COVID-19 [61]. Plasma GFAP is upregulated in activated astrocytes and validated as a biomarker of disease severity [62]. GFAP was increased in acute and mild–moderate COVID-19 individuals who recovered after infection [63]. In our cohort, GFAP levels, starting around age 30, were higher in both Cov and nLongC than in HCs (Figure 8). NFL has been validated to be a biomarker for neuroaxonal damage [64] and also increases with age [34,57,58,59], as well as in acute and mild–moderate COVID-19 [63,65]. In this study, NFL had a stronger correlation with normal aging than GFAP (Figure 8).

HPX is a strong heme-binding protein whose expression increases during an inflammatory response to counter heme-triggered inflammation [66]. HPX also normally increases with age from fetal life to adulthood [67], particularly in the gut (proximal and/or distal colon) [68]. This increase may be linked to age-related changes in gut health. In this study, HPX showed a moderate significant correlation to age for the nLongC group, and weak, insignificant correlations for HC and Cov groups (Figure 8).

In normal or healthy adults, fibrinogen levels tend to increase with age and may contribute to the increased risk of thrombotic events, such as heart attacks and strokes [69]. In our HC group, we did not see a correlation of plasma fibrinogen with age (Figure 8); however, in the Cov and nLongC groups, there were significant correlations. The difference in plasma fibrinogen in nLongC versus Cov is magnified with increasing age, such that the elevation between the two groups is greater in older than in younger individuals, suggesting that abnormally elevated plasma fibrinogen may play a role in contributing to the neurological sequelae seen in some persons post COVID-19 infection. For the IL-8, GFAP, NFL, and fibrinogen studies, the samples included 23 HCs, 35 Cov, and 51 nLongC, and for the HPX assay, 40 HCs, 35 Cov, and 51 nLongC individuals.

### 3.6. Predicting nLongC

One of the objectives of this study was to identify a small number of analytes that could predict nLongC and would return to normal over time. Our data found two striking results: (1) CD14, a monocyte activation marker elevated in nLongC, compared to Cov (Figure 4), and (2) IL-1β could be followed over time to monitor recovery (Figure 7). In addition, both IL-8 and GFAP were significantly elevated in Cov and even more so in nLongC, albeit the increase in nLongC over Cov did not reach statistical significance (Figure 3 and Figure 4). The other marker with significant differences between cohorts was HPX. We used the five genes for the proteins, IL-1β, CXCL8, CD14, GFAP, and HPX, and added APOE to perform GO functional analyses (Table A2). Significant disease classes, categories, biological processes, and clustering included aging, neurological, response to stress, and regulation of immune and defense responses (Table A2). g:Profiler results for WikiPathways included post-COVID-19 neuroinflammation and the COVID-19 adverse outcome pathway (Table A2).

**Figure 9:**
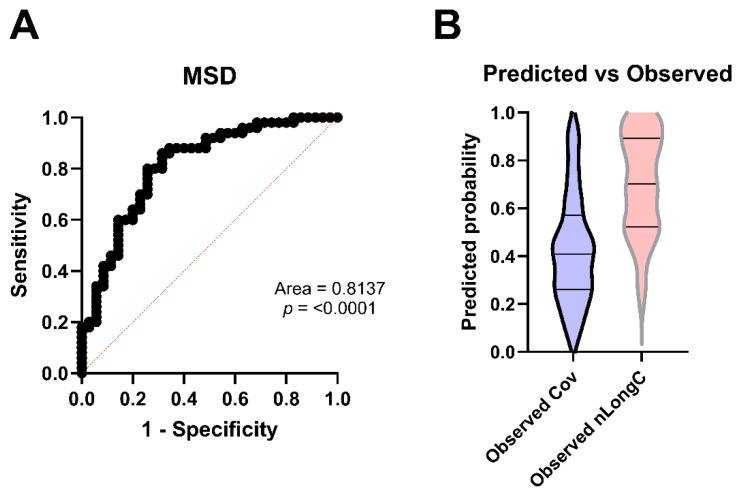
Predicting nLongC. Protein analyses were performed using MSD technology. (**A**) The receiver operating characteristic (ROC) curve and (**B**) the Predicted vs. Observed graph were based on a composite of IL-1β, IL-8, CD14, and GFAP plus age, APOE4, and BMI. Dashed line in panel A represents the ROC curve for the classifier. Horizontal lines in panel B represent quartiles. Group sample sizes were 35 Cov and 50 nLongC.

Using the MSD assay results for IL-1β, IL-8, CD14, and GFAP, along with the presence of the APOE4 gene, and the clinical measurements for age and BMI, we performed multivariate logistic regression using the main effects model to see how well these seven variables predicted nLongC. We did not include HPX, as it was not available on the Ella platform, and we wanted to directly compare our MSD results with the Ella validation results. Using the MSD results, we found an area under the curve (AUC) of 0.8137 (*p* < 0.0001) (Figure 9A). nLongC status was accurately predicted 82.0% of the time, while Cov was 68.6% (Figure 9B and Table A2). The overall percentage of samples that were correctly classified was 76.5% (Table A2).

**Figure 10:**
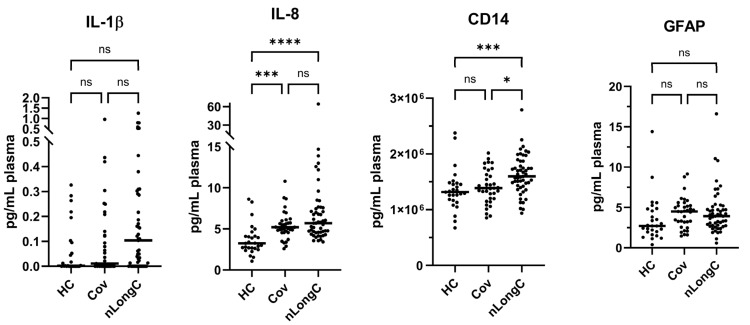
Validation of the MSD manual chemiluminescent ELISA with the Ella automated fluorescent ELISA. Ella data using a custom 4-analyte panel consisting of IL-1β, IL-8, CD14, and GFAP. For all four proteins, group sample sizes were 35 Cov and 51 nLongC. For IL-1β, the HC group size was 26, while for IL-8, CD14, and GFAP, the HC group size was 27. Horizontal bars indicate group medians, which were compared using Kruskal–Wallis tests, followed by post hoc Dunn tests to correct for multiple comparisons. ns = *p* > 0.05, * *p* ≤ 0.05, *** *p* ≤ 0.001, and **** *p* ≤ 0.0001.

For this data to have potential clinical utility, a more facile platform would be important. Therefore, we attempted to recapitulate the above data using a rapid microfluidic technique. Ella (Bio-Techne) is a small benchtop machine that performs automated ELISA in around 90 min using samples loaded onto a microfluidic cartridge. Setup takes only minutes with very little technical expertise required. Of the five proteins that were significantly altered after COVID-19 infection, HPX was not available on the Ella platform. Therefore, we created a custom four-plex panel of analytes containing IL-1β, IL-8, CD14, and GFAP. For these experiments, all 35 Cov and all 51 nLongC participants were included; however, only 26 or 27 HCs were used because of plasma availability. The Ella results (Figure 10) confirmed the IL-8 and CD14 results obtained using the MSD technique (Figure 3 and Figure 4, respectively). Qualitatively, the Ella results for IL-1β and GFAP (Figure 10) were like those obtained by MSD (Figure 3 and Figure 4, respectively). However, quantitatively, the Ella data did not show the statistical differences seen in the MSD results. This discrepancy may be due to the decreased detection sensitivity of the Ella assays and/or smaller sample size of the HC group used in the Ella assays.

**Figure 11:**
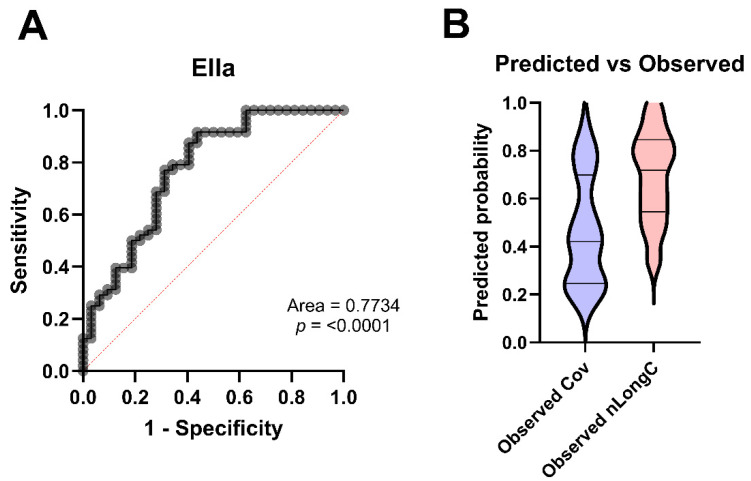
Ella ROC curve and Predicted vs. Observed graph. (**A**) The ROC curve and (**B**) the Predicted vs. Observed graph were based on a composite of IL-1β, IL-8, CD14 and GFAP plus age, APOE and BMI. Dashed line in panel A represents the ROC curve for the classifier. Horizontal lines in panel B represent quartiles. Group sample sizes were 32 Cov and 48 nLongC.

Using the same main effects model and multivariate logistic regression on the results obtained by Ella, we found an AUC of 0.7734 (Figure 11A). Using the Ella data, nLongC status was accurately predicted 83.3% of the time while Cov was 59.4% (Figure 11B and Table A4), with an overall classification accuracy of 73.75% (Table A4). These results resemble those obtained by MSD, thus confirming the validity of these protein analytes as plasma biomarkers.

## 4. Discussion

Global prevalence of LongC often occurs with mild-to-moderate infection [2,3,4]. Cognitive dysfunction continues to be the most frequent symptom, after fatigue, in LongC and often appears later in the illness [5,7]. In this study, we recruited people with nLongC that included new or worsening neurological symptoms or “brain fog” by self-report. We focused our study on peripheral biomarkers that can influence brain function and are associated with inflammation and neuroinflammation.

There was a significant difference in the number of nLongC individuals with an APOE ε4 allele compared to Cov (35% versus 11%). While our numbers are small, this was also reported in a larger cohort [18]. Because APOE4 is the most common genetic risk marker for sporadic AD and other age-related changes such as neuroinflammation and increased blood–brain barrier permeability [70], it was surprising to see that 35% of the nLongC cohort may already be at risk for age-related neurodegeneration simply based on APOE4, even though they were middle-aged at the time of infection.

**Figure A2:**
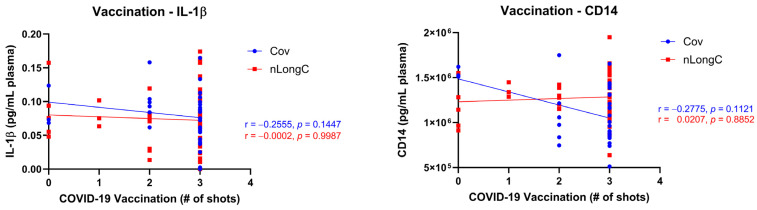
Correlation graphs of COVID-19 vaccination shots with plasma IL-1β and CD14 levels showed no relationships. Group sample sizes were 34 Cov and 51 nLongC. r = Spearman rank correlation coefficient; *p* = *p* value.

Earlier studies have suggested that COVID-19 vaccination may protect against or reduce the effects of LongC, although this is still subject to debate since other studies have shown lack of improvement or worsening of symptoms [71]. A recent study suggested that vaccination prior to infection had no effect on the neurological symptoms of LongC [72]. Consistent with this recent study, our data showed no correlation between the number of COVID-19 shots received by the individual and the plasma levels of IL-1β and CD-14, the two markers we found which distinguished Cov from nLongC (Figure A2).

The risk of cognitive impairment is also greatly influenced by peripheral inflammation and neuroinflammation. Studies of SARS-CoV-2 infection in animals and humans have shown an infiltration of activated monocytes into the brain with microglial activation and an increase in brain IL-1β that decreased neurogenesis and promoted cognitive impairment [73,74]. Underscoring the central role of IL-1β in cognitive impairment is a recent study using a mouse model of acute SARS-CoV-2 infection, which showed not only an infiltration of activated monocytes with microglial activation and an increase in brain IL-1β that decreased neurogenesis and promoted cognitive impairment, but also that vaccination with a low dose of SARS-CoV-2 spike protein prevented IL-1β expression and blocked the subsequent decreased neurogenesis and neurocognitive deficits [75]. These observations are consistent with our results here, showing a significant increase in IL-1β in people with both Cov and nLongC months to years after infection with SARS-CoV-2 compared to pre-COVID-19 healthy controls (Figure 3) and that the recovered Cov cohort had a decrease in IL-1β over time while the nLongC cohort did not (Figure 7). It is possible that the combination of elevated IL-1β plus CD14, an indicator of monocyte and microglia activation seen in individuals with nLongC, may be the drivers of cognitive dysfunction. There are other indicators of brain dysfunction from COVID-19 infection. We previously reported on proteins found in neuronal-enriched extracellular vesicles (nEV) isolated from the plasma of a subset of the Cov and nLongC cohorts presented in this study. Of the nine neurodegenerative proteins tested, pTau181 was significantly elevated in nLongC compared to Cov and HCs [12]. Neurogranin was significantly elevated in both Cov and nLongC over HC, and six other neurotoxic proteins were significantly elevated only in nLongC, again suggesting that people recovered from COVID-19 may have prolonged issues that not only affect the immune system but also likely impact neuronal function.

Normal aging, or any chronic inflammatory condition or insult that causes an increase in chronic immune activation, can increase peripheral inflammation and neuroinflammation, thereby putting people at risk for cognitive impairment [76]. Such an upregulated immune response, which is sterile and low-grade, is a phenomenon known as inflammaging [77]. Since our cohort was middle aged, it was unusual to see that so many of the proteins tested were chronically elevated months to even years after initial infection. This observation that LongC can take months to years to resolve for some people is consistent with the hypothesis that SARS-CoV-2 virus can alter the immune system to cause inflammaging, thereby accelerating biological aging and worsening age-related symptoms and diseases. Our study included people who were not severely ill or hospitalized and were younger with a mean age of 43 years. In addition, symptoms consistent with one getting older, as well as increasing blood markers of inflammation, were more obvious in middle-aged individuals with nLongC. Coupling this with an early report [78] and a recent study [11] showing that younger individuals with COVID-19 infection had a higher risk of neurological complications of LongC points to the possibility that inflammaging is occurring in nLongC individuals, which leads to premature multi-organ pathologies, including the brain [53,79]. Because older individuals had more co-morbidities and more neurologic symptoms prior to COVID-19 infection, inflammaging may not be quite as apparent in these older people. Due to immunosenescence [11,79,80], older individuals with inflammation associated with nLongC may not react anymore or react as much to the stimulus, making COVID-19 infection more problematic for the young and middle-aged than for the old.

Blood–brain barrier integrity decreases with age and is associated with cognitive impairment when modified by other variables, including SARS-CoV-2 [81]. This can allow for an accumulation of neurotoxic proteins and red blood cells (RBCs) into the brain [82,83]. Extravasation of RBCs and direct viral endothelial damage from COVID-19 infection can cause free heme-induced neuroinflammation and cognitive impairment [84,85]. HPX is a heme scavenger protein that increases with age, is significantly elevated in both Cov and nLongC compared to controls (Figure 4), and has been associated with COVID-19 severity [46]. Infection by SARS-CoV-2 virus may require higher levels of HPX to scavenge and reduce the toxic effects of free heme. This may explain why HPX levels are higher in nLongC than in HCs and Cov individuals (Figure 4). The role of HPX in binding heme and its association with cognitive impairment suggest that iron may play an important role in cognition and physiological aging [86]. Indeed, a recent finding showed that an increase in an iron-associated protein, ferritin light chain 1 (FTL1), a pro-aging neuronal protein, was associated with age-related cognitive impairment in mice [87].

Not only are the plasma protein levels different between the Cov and nLongC groups, but we also saw a difference in the rates of change for pro-inflammatory and anti-inflammatory proteins, with pro-inflammatory proteins decreasing faster in Cov and anti-inflammatory proteins decreasing faster in nLongC (Figure A1). Because IL-10 has been shown to dampen inflammation by inhibiting the production of inflammatory agents such as IL-1β and CD14 [88,89,90], it is possible in nLongC individuals, the lack of downregulation of IL-1β over time (Figure 7) coupled with the positive correlation between IL-10 and CD14 (Figure 6) is an indication that the initial acute immune cell activation and inflammation may not be adequately turning off. Similar positive correlations seen here for nLongC have been observed in other neuroinflammatory diseases. In people with virally suppressed HIV infection, the viral accessory protein Nef can activate monocytes, inducing IL-10 production by monocyte-derived macrophages. This leads to increased production of soluble CD14 and CD163 by the macrophages, thereby driving the chronic inflammation seen in that population [91].

One of the objectives of this study was to see if there were different blood analytes that could differentiate people who recover from COVID-19 infection from those who have lingering neurologic symptoms for purposes of diagnosis and monitoring recovery. Of the many proteins reported to be elevated early on in COVID-19 infection and that we tested, only CD14 delineated individuals with Cov from nLongC, while IL-1β decreased over time in Cov but not nLongC many months to years post infection. As proof of concept, we used a panel of four protein markers (IL-1β, CD14, IL-8, and GFAP), found using two different experimental platforms (MSD and Ella), along with APOE4 presence, age, and BMI, to predict nLongC outcome. While the MSD technique was more sensitive, with a better AUC, the Ella platform has the advantage of being rapid and easy to perform. As new blood markers are discovered, a customized panel using the Ella platform is achievable for a clinical laboratory.

There are limitations to this study. One is the small number of samples, although we were successful in validating several of the markers that remain elevated over time [12], as well as other studies that have also found the same prevalence of the APOE4 genotype in nLongC. Another limitation is the uncertainty of the storage and handling of the HC plasma prior to analysis. Unlike the Cov and nLongC plasma samples, which were all collected in house, processed, aliquoted, and frozen immediately prior to use, the HC plasma was purchased from a commercial vendor, and we do not know the details of the processing and handling procedures, which may affect protein stability. Despite this limitation, we chose to use the commercially purchased plasma because the “pre-pandemic” nature of these samples meant that they were likely not exposed to the SARS-CoV-2 virus in contrast to plasma collected post pandemic. A third drawback is that the analytes studied here are not exhaustive; however, they were targeted based on what we and others have previously found to be differentially expressed in brain disorders and/or altered in nLongC. Although we tried to examine the protein levels over time by correlating them with days post infection, this was not a longitudinal study, since group data was used, and the same individuals were not followed over time. In the future, a longitudinal study would be very useful in confirming the IL-1β results, as well as the other pro-inflammatory and anti-inflammatory proteins that appear to differ over time between the groups. Because the brain fog was subjectively reported by the individual and not objectively assessed by the clinician, it may result in errors and inconsistencies. Finally, we recognize that our nLongC group had more comorbidities than the Cov group, which may be a confounder influencing the subjective symptoms and inflammation induced by COVID-19 infection.

Given that dementia may take years of neuroinflammation before clinical manifestations, nLongC has the elements for initiation and progression to cognitive impairment. Even though the symptoms of the participants were subjective, there were blood markers to differentiate those that continued to show cognitive symptoms from those who recover, arguing a true biological cause for “brain fog”. Some of the blood markers showed no difference between the two post-COVID-19 groups over time, suggesting that people who think they are recovered may continue to have blood markers of peripheral and neuroinflammation for a year or more. It also supports the idea of a SARS-CoV-2 reservoir that is driving the inflammation, and the similarities to HIV infection have been reported [92]. Therapeutic targeting of the virus may not be sufficient, as the mechanism or form of persistence has not been proven. Individuals who ultimately recover from LongC may have a healthier immune system, although the younger age of those with LongC might dispute that. Here we report that even people who recover from COVID-19 infection can show chronic neurological and immunological consequences. Our data suggest distinct differences between pre-pandemic, healthy controls, COVID-19-infected individuals who fully recover, and those who continue to suffer neurological sequelae.

## Figures and Tables

**Table 1:** Demographics of healthy control (HC), recovered COVID (Cov), and neuro-long COVID (nLongC) groups.

		HC (% or SD) *n* = 51	Cov (% or SD) *n* = 35	nLongC (% or SD) *n* = 51	*p* Value
**Sex (%)**	Female	17 (33.3)	19 (54.3)	25 (49.0)	0.1150 ^a^
	Male	34 (66.7)	16 (45.7)	26 (51.0)	
**Race (%)**	White	27 (52.9)	14 (40.0)	29 (56.9)	0.0003 ^a,#^
	African American	10 (19.6)	3 (8.6)	9 (17.6)	
	Asian	2 (3.9)	15 (42.9)	6 (11.8)	
	Other	0 (0)	3 (8.6)	4 (7.8)	
	Unknown	12 (23.5)	0 (0)	3 (5.9)	
**Ethnicity (%)**	Hispanic	5 (9.8)	4 (11.4)	9 (17.6)	0.6882 ^a,#^
	Non-Hispanic	38 (74.5)	31 (88.6)	42 (82.4)	
	Unknown	8 (15.7)	0 (0)	0 (0)	
**Apolipoprotein E genotype (%)**	ε2, ε3	NA	5 (14.3)	5 (9.8)	0.1046 ^a^
	ε2, ε4	NA	0 (0)	1 (2.0)	
	ε3, ε3	NA	26 (74.3)	28 (54.9)	
	ε3, ε4	NA	4 (11.4)	16 (31.4)	
	ε4, ε4	NA	0 (0)	1 (2.0)	
	ε4 positive	NA	4 (11.4)	18 (35.3)	0.0135 ^a^
**Comorbidity ^§^ (%)**		NA	3 (5.7)	19 (37.3)	0.0027 ^a^
Vaccination status * (%)	Vn	NA	3 (8.6)	5 (9.8)	0.4387 ^a^
	Vb	NA	4 (11.4)	12 (23.5)	
	Va	NA	28 (80.0)	33 (64.7)	
	Vu	NA	0 (0)	1 (2.0)	
**Multiple infections till visit (%)**		NA	2 (5.7)	2 (3.9)	>0.9999 ^a^
**Days till visit, Mean (SD)**		NA	462 (340)	469 (351)	0.9182 ^b^
**Body Mass Index, Mean (SD)**		NA	24.2 (4.1)	29.2 (20.6)	0.1159 ^b^
**Age in years, Range** **Mean (SD)**		20–76 47.7 (13.1)	18–69 38.1 (16.6)	19–64 42.9 (12.6)	0.0086 ^c^

SD, standard deviation. NA, not available. ^a^ Fisher’s exact test. ^b^ Mann–Whitney test, two-tailed. ^c^ Kruskal–Wallis test followed by Dunn’s post-test. HC vs. Cov (*p* = 0.0069); HC vs. nLongC (*p* = 0.2213); Cov vs. nLongC (*p* = 0.4546). ^#^ Cases with unknown race/ethnicity were excluded from statistical analysis. ^§^ Comorbidities were arthritis, celiac disease, clear cell carcinoma, diabetes, heart problems, Helicobacter pylori infection, hypertension, lung disease, obesity, or vasovagal syncope. * Vn, unvaccinated; Vb, infected before vaccination; Va, infected after vaccination; Vu, vaccinated but vaccination date is unknown; Unknown/undisclosed, vaccine status is unknown or undisclosed.

## Data Availability

The data described in this study is available from the corresponding author upon reasonable request.

## Abbreviations

APOE	Apolipoprotein E
APOE4	Apolipoprotein E ε4 allele
BMI	Body mass index
C3	Complement C3
CD14	(Soluble) Cluster of differentiation 14
CD163	(Soluble) Cluster of differentiation 163
Cov	People fully recovered from SARS-Cov-2 infection with no lingering symptoms >3 months post infection
Days P.I.	Days post infection
GFAP	Glial fibrillary acidic protein
HCs	Healthy controls, pre-pandemic
HPX	Hemopexin
HIV	Human immunodeficiency virus
IL-1β	Interleukin 1 beta
IL-8	Interleukin 8
IL-10	Interleukin 10
LongC	LongCOVID-19
NFL	Neurofilament light chain
nLongC	People with continued neurological sequelae >3 months post SARS-Cov-2 infection
PASC	Post-acute sequelae of COVID-19
RBC	Red blood cell

## Appendix A

**Table A1:** *p* values for Spearman’s correlation coefficients shown in correlation matrices.

	Age	Days P.I.	BMI	Fibrinogen	C3	HPX	IL-1β	IL-8	IL-10	IL-18	GFAP	NFL	CD14	CD163
**HC**														
**Age**				0.984	0.310	0.151	0.914	0.001	0.338	0.142	0.010	0.000	0.784	0.120
**Days P.I.**														
**BMI**														
**Fibrinogen**	0.983899				0.480	0.188	0.540	0.270	0.838	0.333	0.178	0.700	0.309	0.071
**C3**	0.3099399			0.480		5.894 × 10^−7^	0.688	0.054	0.229	0.435	0.048	0.461	0.026	0.748
**HPX**	0.1506363			0.188	5.894 × 10^−7^		0.312	0.132	0.410	0.557	0.414	0.267	0.298	0.749
**IL-1β**	0.914376			0.540	0.688	0.312		0.975	0.305	0.929	0.939	0.491	0.166	0.529
**IL-8**	0.0007487			0.270	0.054	0.132	0.975		0.060	0.346	0.047	0.155	0.807	0.302
**IL-10**	0.3380212			0.838	0.229	0.410	0.305	0.060		0.843	0.247	0.313	0.109	0.438
**IL-18**	0.1419747			0.333	0.435	0.557	0.929	0.346	0.843		1.000	1.000	1.000	1.000
**GFAP**	0.0102523			0.178	0.048	0.414	0.939	0.047	0.247	1.000		0.077	0.394	0.396
**NFL**	0.0001034			0.700	0.461	0.267	0.491	0.155	0.313	1.000	0.077		0.210	0.142
**CD14**	0.7842992			0.309	0.026	0.298	0.166	0.807	0.109	1.000	0.394	0.210		0.000
**CD163**	0.1203823			0.071	0.748	0.749	0.529	0.302	0.438	1.000	0.396	0.142	0.000	
**Cov**														
**Age**		0.387	0.648	0.018	0.789	0.328	0.646	0.087	0.068	0.278	0.002	0.000	0.001	0.096
**Days P.I.**	0.387		0.096	0.485	0.606	0.474	0.005	0.665	0.459	0.555	0.187	0.666	0.411	0.302
**BMI**	0.648	0.096		0.004	2.902 × 10^−6^	0.005	0.065	0.264	0.648	0.002	0.008	0.469	0.749	0.143
**Fibrinogen**	0.018	0.485	0.004		0.006	0.195	0.128	0.697	0.470	0.104	0.715	0.914	0.013	0.008
**C3**	0.789	0.606	2.902 × 10^−6^	0.006		6.254 × 10^−7^	0.055	0.018	0.199	0.046	0.124	0.197	0.929	0.122
**HPX**	0.328	0.474	0.005	0.195	6.254 × 10^−7^		0.511	0.197	0.888	0.007	0.363	0.517	0.672	0.538
**IL-1β**	0.646	0.005	0.065	0.128	0.055	0.511		0.050	0.260	0.124	0.280	0.194	0.381	0.556
**IL-8**	0.087	0.665	0.264	0.697	0.018	0.197	0.050		0.177	0.808	0.360	0.373	0.034	0.432
**IL-10**	0.068	0.459	0.648	0.470	0.199	0.888	0.260	0.177		1.000	0.589	0.124	0.678	0.937
**IL-18**	0.278	0.555	0.002	0.104	0.046	0.007	0.124	0.808	1.000		0.773	0.750	0.492	0.141
**GFAP**	0.002	0.187	0.008	0.715	0.124	0.363	0.280	0.360	0.589	0.773		0.045	0.065	0.743
**NFL**	0.000	0.666	0.469	0.914	0.197	0.517	0.194	0.373	0.124	0.750	0.045		0.544	0.699
**CD14**	0.001	0.411	0.749	0.013	0.929	0.672	0.381	0.034	0.678	0.492	0.065	0.544		0.203
**CD163**	0.096	0.302	0.143	0.008	0.122	0.538	0.556	0.432	0.937	0.141	0.743	0.699	0.203	
**nLongC**														
**Age**		0.024	0.004	0.019	2.308 × 10^−5^	9.902 × 10^−5^	0.061	0.003	0.986	0.009	0.012	9.82 × 10^−8^	0.226	0.686
**Days P.I.**	0.024		0.123	0.937	0.256	0.182	0.395	0.359	0.962	0.680	0.939	0.117	0.899	0.201
**BMI**	0.004	0.123		0.002	7.365 × 10^−6^	0.023	0.942	0.023	0.837	0.031	0.440	0.093	0.945	0.080
**Fibrinogen**	0.019	0.937	0.002		1.888 × 10^−5^	0.001	0.313	0.214	0.396	0.000	0.582	0.076	0.104	0.055
**C3**	0.000	0.256	7.365 × 10^−6^	1.888 × 10^−5^		8.374 × 10^−12^	0.249	0.001	0.039	4.71 × 10^−5^	0.912	0.003	0.205	0.008
**HPX**	0.000	0.182	0.023	0.001	8.374 × 10^−12^		0.340	0.061	0.222	0.001	0.700	0.005	0.011	0.047
**IL-1β**	0.061	0.395	0.942	0.313	0.249	0.340		5.47 × 10^−5^	0.001	0.938	0.987	0.144	0.181	0.731
**IL-8**	0.003	0.359	0.023	0.214	0.001	0.061	5.467 × 10^−5^		0.003	0.591	0.973	0.060	0.336	0.097
**IL-10**	0.986	0.962	0.837	0.396	0.039	0.222	0.001	0.003		0.799	0.638	0.525	0.019	0.000
**IL-18**	0.009	0.680	0.031	0.000	0.000	0.001	0.938	0.591	0.799		0.239	0.020	0.234	0.867
**GFAP**	0.012	0.939	0.440	0.582	0.912	0.700	0.987	0.973	0.638	0.239		0.034	0.553	0.718
**NFL**	9.819 × 10^−8^	0.117	0.093	0.076	0.003	0.005	0.144	0.060	0.525	0.020	0.034		0.554	0.784
**CD14**	0.226	0.899	0.945	0.104	0.205	0.011	0.181	0.336	0.019	0.234	0.553	0.554		0.009
**CD163**	0.686	0.201	0.080	0.055	0.008	0.047	0.731	0.097	0.000	0.867	0.718	0.784	0.009	

## Appendix C

**Table A2:** Gene enrichment and functional annotation analyses using DAVID and g:Profiler.

Category	Term	Genes	Count	*p*-Value	Benjamini
GAD Disease Class	Aging	CXCL8, IL1B, APOE, GFAP	4	0.0035	0.0325
GAD Disease Class	Reproduction	CXCL8, IL1B, CD14, APOE	4	0.0036	0.0325
GAD Disease Class	Pharmacogenomic	HPX, CXCL8, IL1B, CD14, APOE	5	0.0081	0.0358
GAD Disease Class	Neurological	CXCL8, IL1B, CD14, APOE, GFAP	5	0.0101	0.0358
GAD Disease Class	Renal	CXCL8, IL1B, CD14, APOE	4	0.0110	0.0358
GAD Disease Class	Normal Variation	CXCL8, IL1B, APOE	3	0.0146	0.0358
GAD Disease Class	Other	CXCL8, IL1B, CD14, APOE	4	0.0153	0.0358
GAD Disease Class	Unknown	CXCL8, IL1B, CD14, APOE	4	0.0159	0.0358
GAD Disease Class	Vision	CXCL8, IL1B, APOE	3	0.0254	0.0509
GAD Disease Class	Infection	CXCL8, IL1B, CD14, APOE	4	0.0353	0.0636
**Category**	**Term**	**Genes**	**Count**	***p*-Value**	**Benjamini**
GOTERM BP All	Regulation of response to external stimulus	HPX, CXCL8, IL1B, CD14, APOE	5	0.0001	0.0369532
GOTERM BP All	Response to stress	HPX, CXCL8, IL1B, CD14, APOE, GFAP	6	0.0002	0.0369532
GOTERM BP All	Regulation of signaling	HPX, CXCL8, IL1B, CD14, APOE, GFA	6	0.0003	0.0369532
GOTERM BP All	Regulation of cell communication	HPX, CXCL8, IL1B, CD14, APOE, GFAP	6	0.0003	0.0369532
GOTERM BP All	Regulation of response to biotic stimulus	HPX, IL1B, CD14, APOE	4	0.0003	0.0369532
GOTERM BP All	Biological process involved in interspecies interaction between organisms	HPX, CXCL8, IL1B, CD14, APOE	5	0.0003	0.0369532
GOTERM BP All	Positive regulation of response to external stimulus	HPX, CXCL8, IL1B, CD14	4	0.0003	0.0369532
GOTERM BP All	Regulation of immune process	HPX, CXCL8, IL1B, CD14, APOE	5	0.0003	0.0369532
GOTERM BP All	Positive regulation of multicellular organismal process	CXCL8, IL1B, CD14, APOE, GFAP	5	0.0003	0.0369532
GOTERM BP All	Positive regulation of cell communication	HPX, IL1B, CD14, APOE, GFAP	5	0.0004	0.0386044
GOTERM BP All	Positive regulation of cell signaling	HPX, IL1B, CD14, APOE, GFAP	5	0.0004	0.0386044
GOTERM BP All	Import into cell	CXCL8, CD14, APOE, GFAP	4	0.0005	0.0410767
**Annotation Cluster 1**	**Enrichment Score: 2.8**	**Genes**	**Count**	***p*-Value**	**Benjamini**
GOTERM BP All	Regulation of response to biotic stimulus	HPX, IL1B, CD14, APOE	4	0.0003	0.0370
GOTERM BP All	Regulation of defense response	HPX, IL1B, CD14, APOE	4	0.0010	0.0663
GOTERM BP All	Regulation of immune response	HPX, IL1B, CD14, APOE	4	0.0016	0.0748
GOTERM BP All	Regulation of response to stress	HPX, IL1B, CD14, APOE	4	0.0043	0.1161
GOTERM BP All	Positive regulation of signal transduction	HPX, IL1B, CD14, APOE	4	0.0057	0.1200
**Annotation Cluster 2**	**Enrichment Score: 2.66**	**Genes**	**Count**	***p*-Value**	**Benjamini**
GOTERM BP All	Positive regulation of multicellular organismal process	CXCL8, IL1B, CD14, APOE, GFAP	5	0.0003	0.0370
GOTERM BP All	Regulation of multicellular organismal process	CXCL8, IL1B, CD14, APOE, GFAP	5	0.0032	0.1135
GAD Disease Class	Neurological	CXCL8, IL1B, CD14, APOE, GFAP	5	0.0101	0.0358
**Source**	**Term Name**	**Genes**	**Count**	**Padj**	
WP	LTF danger signal response pathway	CD14, CXCL8, IL1B	3	8.357 × 10^−6^	
WP	Post COVID neuroinflammation	CXCL8, GFAP, IL1B	3	1.526 × 10^−5^	
WP	Toll like receptor signaling	CD14, CXCL8, IL1B	3	1.178 × 10^−3^	
WP	Spinal cord injury	CXCL8, GFAP, IL1B	3	2.094 × 10^−3^	
WP	COVID 19 adverse outcome pathway	CXCL8, IL1B	2	2.632 × 10^−3^	
WP	LDL influence on CD14 and TLR4	CD14, IL1B	2	6.329 × 10^−3^	
WP	Antiviral and anti inflammatory effects on Nrf2 on SARS CoV 2 pathway	CXCL8, IL1B	2	1.238 × 10^−2^	
WP	Prostaglandin signaling	CXCL8, IL1B	2	1.318 × 10^−2^	
WP	Photodynamic therapy induced NF kB survival signaling	CXCL8, IL1B	2	1.484 × 10^−2^	
WP	Immune infiltration in pancreatic cancer	CXCL8, IL1B	2	1.847 × 10^−2^	
WP	Network map of SARS CoV 2 signaling	CD14, CXCL8, IL1B	3	2.208 × 10^−2^	
WP	IL26 signaling	CXCL8, IL1B	2	2.576 × 10^−2^	
WP	Vitamin B12 metabolism	APOE, IL1B	2	3.554 × 10^−2^	
WP	Lung fibrosis	CXCL8, IL1B	2	4.841 × 10^−2^	

## Appendix D

**Table A3:** Multivariate logistic regression analysis for nLongC: MSD data set.

Parameter Estimates	Variable	Estimate	Standard Error	95% CI (Profile Likelihood)
β0	Intercept	−6.152	2.648	−12.03 to −1.506
β1	Age	−0.001986	0.023	−0.04825 to 0.04290
β2	ApoE4[Yes]	1.241	0.7098	−0.07853 to 2.761
β3	BMI	0.1129	0.06046	0.008530 to 0.2432
β4	IL-1β	−8.766	8.521	−26.62 to 7.141
β5	IL-8	0.1661	0.2802	−0.3453 to 0.7678
β6	GFAP	0.01614	0.03287	−0.03937 to 0.09608
β7	CD14	1.367 × 10^−6^	1.832 × 10^−6^	−2.289 × 10^−6^ to 5.006 × 10^−6^
β8	CD14/IL-1β	7.482 × 10^−9^	3.143 × 10^−8^	−5.655 × 10^−8^ to 6.792 × 10^−8^
β9	IL-8/IL-1β	−0.0102	0.0174	−0.04457 to ???
β10	GFAP/IL-1β	0.0007072	0.0007783	3.462 × 10^−5^ to 0.002890
β11	CD14/IL-8	1.176 × 10^−6^	4.248 × 10^−6^	−6.629 × 10^−6^ to 1.039 × 10^−5^
β12	CD14/GFAP	2.520 × 10^−5^	3.204 × 10^−5^	−2.951 × 10^−5^ to 0.0001009
**Odds ratios**	**Variable**	**Estimate**		**95% CI (profile likelihood)**
β0	Intercept	0.002129		5.969 × 10^−6^ to 0.2219
β1	Age	0.998		0.9529 to 1.044
β2	ApoE4[Yes]	3.461		0.9245 to 15.82
β3	BMI	1.119		1.009 to 1.275
β4	IL-1β	0.000156		2.745 × 10^−12^ to 1262
β5	IL-8	1.181		0.7080 to 2.155
β6	GFAP	1.016		0.9614 to 1.101
β7	CD14	1.000		1.000 to 1.000
β8	CD14/IL-1β	1.000		1.000 to 1.000
β9	IL-8/IL-1β	0.9899		0.9564 to ???
β10	GFAP/IL-1β	1.001		1.000 to 1.003
β11	CD14/IL-8	1.000		1.000 to 1.000
β12	CD14/GFAP	1.000		1.000 to 1.000
**Area under the ROC curve**				
Area	0.8137			
Std. Error	0.04798			
95% confidence interval	0.7197 to 0.9078			
*p* value	<0.0001			
**Classification table**	**Predicted Cov**	**Predicted nLongC**	**Total**	**% Correctly classified**
Observed Cov	24	11	35	68.57
Observed nLongC	9	41	50	82
Total	33	52	85	76.47
Negative predictive power (%)	72.73			
Positive predictive power (%)	78.85			
Classification cutoff	0.5			
**Data summary**				
Number of nLongC	50			
Number of Cov	35			

**Table A4:** Multivariate logistic regression analysis for nLongC: Ella data set.

Parameter Estimates	Variable	Estimate	Standard Error	95% CI (Profile Likelihood)
β0	Intercept	−5.537	2.727	−11.41 to −0.7910
β1	Age	0.01469	0.02542	−0.03554 to 0.06524
β2	ApoE4[Yes]	0.91	0.7308	−0.4703 to 2.457
β3	BMI	0.07305	0.05881	−0.01032 to 0.1962
β4	IL-1b	−0.2131	1.327	−2.795 to 2.720
β5	IL-8	0.2633	0.316	−0.07788 to 0.9661
β6	GFAP	0.009413	0.2191	−0.3920 to 0.5003
β7	sCD14	2.258 × 10^−7^	1.706 × 10^−6^	−3.229 × 10^−6^ to 3.428 × 10^−6^
β8	CD14/IL-1b	6.73 × 10^−11^	8.565 × 10^−10^	−1.610 × 10^−9^ to 1.855 × 10^−9^
β9	IL-8/IL-1b	−0.001359	0.0008232	−0.003074 to 0.0002059
β10	sGFAP/IL-1b	0.0009888	0.000924	−0.001090 to 0.002764
β11	CD14/IL-8	3.416 × 10^−6^	7.709 × 10^−6^	−9.011 × 10^−6^ to 1.953 × 10^−5^
β12	CD14/GFAP	1.607 × 10^−6^	1.839 × 10^−6^	−1.038 × 10^−6^ to 6.298 × 10^−6^
**Odds ratios**	**Variable**	**Estimate**		**95% CI (profile likelihood)**
β0	Intercept	0.003939		1.105 × 10^−5^ to 0.4534
β1	Age	1.015		0.9651 to 1.067
β2	ApoE4[Yes]	2.484		0.6248 to 11.67
β3	BMI	1.076		0.9897 to 1.217
β4	IL-1b	0.808		0.06110 to 15.18
β5	IL-8	1.301		0.9251 to 2.628
β6	GFAP	1.009		0.6757 to 1.649
β7	sCD14	1.000		1.000 to 1.000
β8	CD14/IL-1b	1.000		1.000 to 1.000
β9	IL-8/IL-1b	0.9986		0.9969 to 1.000
β10	sGFAP/IL-1b	1.001		0.9989 to 1.003
β11	CD14/IL-8	1.000		1.000 to 1.000
β12	CD14/GFAP	1.000		1.000 to 1.000
**Area under the ROC curve**				
Area	0.7734			
Std. Error	0.05519			
95% confidence interval	0.6653 to 0.8816			
*p* value	<0.0001			
**Classification table**	**Predicted Cov**	**Predicted nLongC**	**Total**	**% Correctly classified**
Observed Cov	19	13	32	59.38
Observed nLongC	8	40	48	83.33
Total	27	53	80	73.75
Negative predictive power (%)	70.37			
Positive predictive power (%)	75.47			
Classification cutoff	0.5			
**Data summary**				
Number of nLongC	48			
Number of Cov	32			
